# 云南省昆明市城市居民肺癌筛查结果分析

**DOI:** 10.3779/j.issn.1009-3419.2019.07.02

**Published:** 2019-07-20

**Authors:** 艳苹 林, 洁 马, 俊 封, 强 张, 云超 黄

**Affiliations:** 650118 昆明，云南省肿瘤医院/昆明医科大学第三附属医院/云南省癌症中心 Yunnan Cancer Hospital/The Third Affiliated Hospital of Kunming Medical University/Yunnan Cancer Center, Kunming 650118, China

**Keywords:** 肺肿瘤, 肿瘤筛查, 危险因素, Lung neoplasms, Cancer screening, Risk factors

## Abstract

**背景与目的:**

肺癌筛查是降低肺癌死亡率的有效措施。目前国际及全国范围内均推荐肺癌筛查，我国于2009年、2012年分别启动了大规模的农村、城市肺癌早诊早治项目，云南省先后于2009年、2014年参与相关项目，但目前云南省尚未报道大规模肺癌筛查的研究结果。本研究通过参加国家癌症中心牵头的城市癌症早诊早治项目，分析2014年-2018年云南省昆明市10, 154例城市居民肺癌筛查结果，评价城市癌症早诊早治肺癌筛查效果。

**方法:**

以云南省昆明市40岁-74岁居民为研究对象，通过防癌风险问卷调查评估出肺癌高风险31, 824人，对高风险人群进行胸部低剂量螺旋计算机断层扫描（computed tomography, CT），分析肺癌的高风险率、筛查率和检出率。

**结果:**

5年完成问卷调查150, 535人，评估出肺癌高危人群31, 824人，高风险率21.14%，共完成胸部低剂量螺旋CT筛查10, 154人，参与率31.91%。肺内结节检出率22.28%（2, 262/10, 154），其中 < 5 mm实性/部分实性结节检出率11.30%（1, 332/10, 154）、 < 8 mm非实性结节检出率2.20%（219/10, 154）、阳性结节检出率7.00%（711/10, 154）、疑似肺癌检出率0.60%（59/10, 154），肺癌检出率0.27%（27/10, 154）。男性阳性结节、 < 8 mm非实性结节及疑似肺癌检出率显著高于女性；阳性结节、 < 5 mm实性结节、疑似肺癌及确诊肺癌检出率随年龄增加而增高，60岁以上年龄组最高。

**结论:**

低剂量螺旋CT适合肺癌高危人群筛查，有助于早期发现肺内的阳性结节和相关疾病，应重视60岁以上人群肺癌的筛查工作；了解了昆明市居民的肺部健康状况，提高了居民的肺癌防治意识，提高肺癌的早诊早治率。

## Abstract (en)

**Background and objective:**

Lung cancer screening is an effective measure to reduce lung cancer mortality. Currently, lung cancer screening is recommended internationally and nationally. China had launched a large-scale early diagnosis and early treatment of rural and urban lung cancer in 2009 and 2012. Yunnan Province participated in related projects in 2009 and 2014, but the results of large-scale lung cancer screening have not been reported in Yunnan Province. In this study, we participated in the National Key Public Health Program, Cancer Screening Program in Urban China and analyzed the results of lung cancer screening in 10, 154 urban residents in Kunming, Yunnan Province from 2014 to 2018, and evaluated the screening effect of early diagnosis and treatment of lung cancer in urban.

**Methods:**

A total of 31, 824 people with high risk of lung cancer were evaluated with 40-74 year-old population in Kunming. A high-risk group underwent low-dose spiral computed tomography (CT) scan of the chest, and analyze the high risk rate and detection rate. A total of 150, 535 questionnaires were completed in 5 years, and 31, 824 people with high risk of lung cancer were evaluated.

**Results:**

The high-risk rate was 21.14%. A total of 10, 154 low-dose spiral CT screenings were performed, with a participation rate of 31.91%. The detection rate of intrapulmonary nodules was 22.28% (2, 262/10, 154), of which the detection rate of < 5 mm solid/partial solid nodules was 11.30% (1, 332/10, 154) and the detection rate of < 8 mm non-solid nodules was 2.20% (219/10, 154). The positive nodule detection rate was 7.00% (711/10, 154), the suspected lung cancer detection rate was 0.60% (59/10, 154), and the lung cancer detection rate was 0.27% (27/10, 154). The detection rate of the positive nodules, < 8 mm non-solid nodules and suspected lung cancer in male were significantly higher than that in female, and the positive nodules, < 5 mm solid nodules, suspected lung cancer and confirmed lung cancer detection rate increased with age.

**Conclusion:**

Low-dose spiral CT is suitable for screening of high-risk populations of lung cancer, which is helpful for early detection of positive nodules and related diseases in the lungs. It is necessary to pay attention to the screening of lung cancer in people over 60 years old. Understand the lung health status of residents in Kunming. Raise awareness of lung cancer prevention and treatment among residents, and improve the rate of early diagnosis and treatment of lung cancer.

2018年全球癌症数据发布，肺癌仍居全球癌症发病和死亡的首位^[1]^，自21世纪初期，肺癌已成为我国发病率和死亡率最高的恶性肿瘤^[2]^。2015年，我国癌症生存分析数据显示肺癌的发病和死亡例数分别达733, 300人和610, 200人，发病率和死亡率非常接近，其主要原因是晚期病例占比较大，无法手术治疗，预后极差，5年生存率仅为16.1%^[3]^。

2011年，美国国家肺癌筛查随机对照试验结果显示，在高危人群中，与胸部X线片比较，低剂量螺旋计算机断层扫描（low-dose computed tomography, LDCT）可降低20%的肺癌死亡率，首次证实进行LDCT肺癌筛查的获益^[4]^。因此，肺癌的早期诊断和早期治疗是提高肺癌生存、降低肺癌死亡率的重要措施。我国于2009年在国家医改重大专项“农村癌症早诊早治”项目中将肺癌纳入试点，启动了我国肺癌高危人群筛查工作^[5]^。2012年，国家在全国多个省份开展重大公共卫生服务项目“城市癌症早诊早治项目”，启动了我国城市肺癌高危人群筛查工作。云南省昆明市作为第二批项目开展省份，自2014年参加项目已实施开展5年，现将5年的肺癌筛查结果分析如下。

## 1 材料与方法

### 1.1 研究对象

2014年-2018年采用整群抽样的方法，先后纳入昆明市西山区、官渡区、五华区、呈贡区共36个街道的常住居民，纳入标准：（1）本市户籍常驻人口或本地居住3年及以上；（2）入组时实足年龄在40岁-74岁；（3）愿意且能够签署知情同意书；排除标准：研究对象若符合以下任一项条件即排除：（1）无法完成知情同意书；（2）需要氧供应才能保证呼吸功能；（3）仰卧时不能把胳膊放在头上；（4）任何医学原因显示在4年研究期间有显著的死亡风险；（5）胸部或背部有金属植入物或金属设备（心脏起搏器等），影响肺部成像；（6）有癌症史；（7）以前切除过部分肺叶，不包括经皮肺穿刺；（8）有肺癌症状，包括不明原因的体质量减少（过去12个月内体质量减少7.5 kg以上）或不明原因的咳血。

### 1.2 研究方法

#### 1.2.1 危险因素问卷调查

所有调查对象在各街道社区专人指导下自行填写危险因素调查问卷或由经过专业培训的调查员询问调查对象后填写问卷。危险因素调查问卷包括基本信息、饮食习惯、生活环境、生活方式和习惯、心理和情绪、疾病既往史、恶性肿瘤家族史、女性生理和生育史等。

#### 1.2.2 高危人群评估

工作人员质控调查问卷后录入国家癌症中心开发的高危人群评估系统初筛肺癌高危人群。该系统以“哈佛癌症风险指数”^[6]^理论为基础，依据近20年来我国常见癌症流行病学资料，通过多学科专家小组讨论达成共识的方法，确定我国成年人癌症发病的主要危险因素及相关赋值，应用哈佛癌症风险指数工作小组推荐的公式，研发出的适合我国人群的个体癌症风险综合评价体系^[7]^。城市癌症早诊早治项目高危风险评估系统引入肺癌相关因素为吸烟指数（包括是否达到戒烟标准）、日常平均新鲜蔬菜摄入量、长期生活环境空气污染、日常体育锻炼情况、慢性呼吸系统疾病史、肺癌家族史、被动吸烟史等。直入条件为吸烟指数≥400（每日吸烟的支数×吸烟的年数），且年龄≥50岁；超过20年接受二手烟（被动吸烟）者；有较长期职业暴露史者（石棉、铍、铀、氡等）。

#### 1.2.3 临床筛查

问卷调查后评估为肺癌高风险人群至云南省肿瘤医院进行胸部LDCT扫描，扫描厚5 mm，层间距5 mm，重建层厚1.0 mm-1.25 mm连续（层间隔为0），扫描范围从肺尖到肋膈角（包括全部肺），受检者吸气末一次屏气完成扫描。由高年资（3年以上）放射科医师出具检查报告并填写筛查记录表。

#### 1.2.4 进一步治疗及随访

< 5 mm的实性/部分实性结节或 < 8 mm的非实性结节及阴性筛查者间隔12个月复查；5 mm-14 mm的实性/部分实性结节和8 mm-14 mm非实性结节间隔3个月复查；≥15 mm的结节建议抗炎治疗并间隔1个月复查。疑似肺癌建议进一步活检确诊、手术等相关临床治疗。

#### 1.2.5 相关指标定义

① 阳性结节：实性/部分实性结节平均径≥5 mm或非实性结节≥8 mm或检出气管腔内结节；②高风险率=评估为肺癌高风险例数/问卷调查数×100%，筛查参与率（筛查率）=临床检查例数/评估为肺癌高风险例数×100%，检出率=阳性病变例数/临床检查例数×100%；③早期肺癌：Ⅰ期和Ⅱ期肺癌病例；④早诊率=早期肺癌病例数/确诊肺癌病例数。

### 1.3 统计学分析

应用SPSS 19.0统计软件建立数据库及统计分析，计数资料使用卡方检验，计量资料采用*t*检验，*P* < 0.05认为有统计学差异。

## 2 结果

### 2.1 危险因素问卷调查结果

2014年-2018年度，共计150, 535人完成肺癌危险因素评估，评估出高风险31, 824人，总体高风险率为21.14%，男性高风险率为30.16%，女性高风险率为13.43%，且男性高风险率高于女性（*P* < 0.001）；不同年龄组高风险率不同（*P* < 0.001），见表 1。

**1:** 肺癌高风险人群性别、年龄分布 Distribution of the population with lung cancer high risk

Items	Questionnaires (*n*)	High risk population (*n*)	High risk rate (%)	*χ*^2^	*P*
Gender				6, 275.96	< 0.001
Male	69, 384	20, 924	30.16		
Female	81, 151	10, 900	13.43		
Age groups (yr)				633.57	< 0.001
40-	54, 682	10, 846	19.83		
50-	49, 293	12, 264	24.88		
60-	46, 560	8, 714	18.72		
Total	150, 535	31, 824	21.14		

### 2.2 低剂量螺旋CT筛查依从性

31, 824人高风险人群中仅有10, 154人完成了低剂量CT筛查，参与率仅为31.91%，男性参与率24.25%，女性参与率46.61%，女性参与率明显高于男性（χ^2^=1, 648.59, *P* < 0.001）；总体平均年龄（54.30±7.82）岁，男性平均年龄（54.83±7.86）岁，女性平均年龄（53.76±7.74）岁（*P* < 0.001）。不同年龄组低剂量CT筛查参与率不同，50岁-59岁年龄组参与率最高，为33.46%（*P* < 0.001），见表 2。

**2:** 接受LDCT筛查人群的性别、年龄分布 Gender and age distribution of receiving LDCT screening population

Items	High risk population (*n*)	Screening population (*n*)	Participation rate (%)	*χ*^2^	*P*
Gender				1, 648.59	< 0.001
Male	20, 924	5, 074	24.25		
Female	10, 900	5, 080	46.61		
Age groups (years)				65.88	< 0.001
40-	10, 846	3, 144	28.99		
50-	12, 264	4, 104	33.46		
60-	8, 716	2, 906	33.34		
Total	31, 824	10, 154	31.91		
LDCT: low-dose computed tomography.					

### 2.3 低剂量螺旋CT筛查结果

#### 2.3.1 总体结果

胸部LDCT肺内结节检出率22.28%（2, 262/10, 154），其中阳性结节检出率7.00%（711/10, 154）， < 5 mm实性/部分实性结节检出率11.30%（1, 332/10, 154）、 < 8 mm非实性结节检出率2.20%（219/10, 154）。疑似肺癌检出率0.60%（59/10, 154），随访所有疑似肺癌者，成功随访病例中肺癌检出率0.27%（27/10, 154），其中Ⅰ期19例，Ⅱ期5例，Ⅲ期2例，Ⅳ期1例，早诊率为88.89%（24/27）。

#### 2.3.2 性别组间差异分析

男性阳性结节、 < 8 mm非实性结节及疑似肺癌检出率显著高于女性（χ_1_^2^=15.56, *P*_1_ < 0.001; χ_2_^2^=8.58, *P*_2_=0.003; χ_3_^2^=17.13, *P*_3_=0.001）； < 5 mm实性结节检出率和确诊肺癌检出率在性别间的差异无统计学意义（χ_4_^2^=2.99, *P*_4_=0.08; χ_5_^2^=0.038, *P*_5_=0.85）（表 3）。（备注：χ_1_^2^、*P*_1_：阳性结节检出率性别差异统计分析χ^2^值及*P*值；χ_2_^2^、*P*_2_： < 8 mm非实性结节检出率性别差异统计分析χ^2^值及*P*值；χ_3_^2^、*P*_3_：疑似肺癌检出率性别差异统计分析χ^2^值及*P*值；χ_4_^2^、*P*_4_： < 5 mm实性结节检出率性别差异统计分析χ^2^值及*P*值；χ_5_^2^、*P*_5_： < 确诊肺癌检出率性别差异统计分析χ^2^值及*P*值。）

**3:** 肺部结节和肺癌检出汇总表 Detection rate of pulmonary nodules and lung cancer

Items	Positive nodules			< 5 mm solid /partial solid nodules			< 8 mm non-solid nodules			Suspicious lung cancer			Confirmed lung cancer	
	*n*	Detection rate (%)		*n*	Detection rate (%)		*n*	Detection rate (%)		*n*	Detection rate (%)		*n*	Detection rate (%)
Gender														
Male	406	8.00		695	13.70		88	1.73		30	0.59		14	0.28
Female	305	6.00		637	12.54		131	2.58		29	0.57		13	0.26
Age groups (years)														
40-	170	5.41		383	12.18		72	2.29		8	0.25		3	0.10
50-	270	6.58		522	12.27		72	1.75		18	0.43		8	0.19
60-	271	9.33		427	14.69		75	2.58		33	1.14		16	0.55
Total	711	7.00		1, 332	13.10		219	2.20		59	0.60		27	0.27

#### 2.3.3 年龄组间差异分析

阳性结节、 < 5 mm实性结节、疑似肺癌及确诊肺癌检出率随年龄增加而增高，60岁以上年龄组最高，组间差异具有统计学意义（χ_6_^2^=37.50, *P*_6_ < 0.001; χ_7_^2^=9.32, *P*_7_=0.009; χ_8_^2^=48.88, *P*_8_ < 0.001; χ_9_^2^=13.11, *P*_9_=0.001）， < 8 mm非实性结节检出率在各年龄组间的差异无统计学意义（χ_10_^2^=5.89, *P*_10_=0.053）（表 3）。（备注：χ_6_^2^、*P*_6_：阳性结节检出率年龄组间差异统计分析χ^2^值及*P*值；χ_7_^2^、*P*_7_： < 5 mm实性结节检出率年龄组间差异统计分析χ^2^值及*P*值；χ_8_^2^、*P*_8_：疑似肺癌检出率年龄组间差异统计分析χ^2^值及*P*值；χ_9_^2^、*P*_9_： < 确诊肺癌检出率年龄组间差异统计分析χ^2^值及*P*值；χ_10_^2^、*P*_10_： < 8 mm非实性结节检出率年龄组间差异统计分析χ^2^值及*P*值。）

### 2.4 LDCT肺癌筛查参与率及检出率趋势分析

趋势分析显示，2014年-2018年间LDCT参与率呈逐年上升趋势（*P* < 0.001）；阳性结节和肺癌检出人数虽然逐年增多，但其检出率趋势分析无统计学差异（*P* > 0.05），详见表 4。

**4:** 2015年-2018年LDCT参与率及检出率趋势分析 LDCT participation rate and detection rate trend analysis in 2015-2018

Year	LDCT participation rate	Detection rate of positive nodules	Detection rate of confirmed lung cancer
2014-2015	16.67% (1, 157/6, 941)	11.06% (128/1, 157)	0.26% (3/1, 157)
2015-2016	35.11% (3, 187/9, 077)	4.93% (157/3, 187)	0.13% (4/3, 187)
2016-2017	34.77% (2, 721/7, 825)	7.02% (207/2, 721)	0.29% (8/2, 721)
2017-2018	38.70% (3, 089/7, 981)	7.61% (295/3, 089)	0.39% (12/3, 089)
*χ*^2^	682.68	0.12	2.58
*P*	< 0.001	0.73	0.11

## 3 讨论

最新肿瘤登记资料^[8]^显示，云南省居民恶性肿瘤发病率及死亡率首位均为肺癌，是严重危害我省居民健康的恶性肿瘤之一。本研究肺癌高风险评估提示昆明市城市居民肺癌高危人群总体高风险率21.14%，略高于重庆市^[9]^的19.80%；男性高风险率30.16%，女性高风险率13.43%，且男性高风险率高于女性，这可能与男性吸烟率高于女性有关。本次筛查中肺癌高危人群进行LDCT筛查的参与率为31.91%，高于北京市报道的24.76%^[10]^和乌鲁木齐市的29.7%^[11]^，但略低于重庆市报道的37.10%^[9]^。男性筛查依从性低于女性，与乌鲁木齐市^[11]^、重庆市报道一致^[9]^。但总体上肺癌筛查参与率不高，表明居民的肺癌防治意识不高，需加强健康教育宣传，提高居民癌症防治知识知晓率及早诊早治意识。

本研究阳性结节、疑似肺癌的检出率略低于全国^[12]^的8.46%、0.96%及重庆市^[9]^的12.91%、1.59%。不同地区检出率的差异可与各地区肺癌发病特点及诊疗水平不一。本研究发现男性阳性结节、 < 8 mm非实性结节及疑似肺癌检出率显著高于女性，这与其他报道^[9]^一致，可能与男性肺癌发病率高于女性^[2]^及男性吸烟率较高和其他不良生活习惯等肺癌患病高危因素的暴露有关。阳性结节、 < 5 mm实性结节、疑似肺癌及确诊肺癌检出率随年龄增加呈上升趋势，60岁以上人群是阳性结节及肺癌的高发年龄组。年度趋势分析显示，2014年-2018年间LDCT参与率呈逐年上升趋势，阳性结节和肺癌检出人数虽然逐年增多，但其检出率趋势分析无统计学差异。2014年-2015年为城癌项目启动的第一年，相关的检查技术及项目开展的整体经验不足可能是导致当年的参与率及检出率偏低的主要原因。LDCT参与率逐年升高可能与项目开展过程中肿瘤防治知识的大力宣传和居民肿瘤防治意识提高有关；阳性结节及肺癌确诊人数增加可能与筛查技术不断完善，高危人群评估的准确性及可靠性增加以及项目开展相关经验的不断积累有关。可见，癌症筛查项目不但能提高早诊早治率，还能提高居民对肿瘤防治知识的知晓率并促进其参加肿瘤相关筛查、健康体检。

探索适合我国国情的肺癌筛查方案是亟需解决的问题，周清华教授等中国肺癌早诊早治专家组于2015年制定了中国低剂量螺旋CT肺癌筛查指南（2015版）^[13]^，2018年修订了中国低剂量螺旋CT肺癌筛查指南（2018版）^[14]^。如何精确识别、筛选高危人群，如何寻找LDCT筛查出的肺部结节的最佳管理策略，是提高肺癌LDCT筛查获益、减少潜在危害的关键环节，但目前的研究尚无定论，缺乏大规模、多中心的前瞻性研究证据。目前，我国在全国多省市开展农村、城市肺癌筛查项目，并不断设计、完善前瞻性的随机对照筛查研究计划，同时启动了相关生物样本、生物标记物的研发和验证，期待通过项目的开展能够进一步完善肺癌筛查技术方案。
